# Writing 3D Nanomagnets Using Focused Electron Beams

**DOI:** 10.3390/ma13173774

**Published:** 2020-08-26

**Authors:** Amalio Fernández-Pacheco, Luka Skoric, José María De Teresa, Javier Pablo-Navarro, Michael Huth, Oleksandr V. Dobrovolskiy

**Affiliations:** 1 SUPA, School of Physics and Astronomy, University of Glasgow, Glasgow G12 8QQ, UK; 2 Cavendish Laboratory, University of Cambridge, JJ Thomson Avenue, Cambridge CB3 0HE, UK; ls604@cam.ac.uk; 3 Instituto de Nanociencia y Materiales de Aragón (INMA), Universidad de Zaragoza-CSIC, 50009 Zaragoza, Spain; 4 Laboratorio de Microscopías Avanzadas (LMA) and Departamento de Física de la Materia Condensada, Universidad de Zaragoza, 50009 Zaragoza, Spain; j.pablo-navarro@hzdr.de; 5 Institute of Ion Beam Physics and Materials Research, Helmholtz-Zentrum Dresden-Rossendorf, 01328 Dresden, Germany; 6 Institute of Physics, Goethe University Frankfurt, 60438 Frankfurt am Main, Germany; oleksandr.dobrovolskiy@univie.ac.at; 7 Faculty of Physics, University of Vienna, 1090 Vienna, Austria

**Keywords:** nanomagnetism, nanofabrication, 3D printing, additive manufacturing, focused electron beam, lithography, spintronics, magnetic nanowires

## Abstract

Focused electron beam induced deposition (FEBID) is a direct-write nanofabrication technique able to pattern three-dimensional magnetic nanostructures at resolutions comparable to the characteristic magnetic length scales. FEBID is thus a powerful tool for 3D nanomagnetism which enables unique fundamental studies involving complex 3D geometries, as well as nano-prototyping and specialized applications compatible with low throughputs. In this focused review, we discuss recent developments of this technique for applications in 3D nanomagnetism, namely the substantial progress on FEBID computational methods, and new routes followed to tune the magnetic properties of ferromagnetic FEBID materials. We also review a selection of recent works involving FEBID 3D nanostructures in areas such as scanning probe microscopy sensing, magnetic frustration phenomena, curvilinear magnetism, magnonics and fluxonics, offering a wide perspective of the important role FEBID is likely to have in the coming years in the study of new phenomena involving 3D magnetic nanostructures.

## 1. Introduction

Controlling the composition, structure and shape of materials at the nanoscale has become one of the forefronts of modern science, opening new opportunities in virtually all areas of technology. In nanomagnetism, accessing these length scales via 2D patterning and engineering of interfaces has proven to be key for the uncovering of a plethora of fascinating effects including the Giant Magnetoresistance [1], Spin-Transfer and Spin-Orbit Torques and domain wall and skyrmion devices [2,3,4], just to name a few. Owing to the recent advances in computation, fabrication and characterization tools, and driven by fundamental bottlenecks in 2D nanomagnetism, the field of 3D nanomagnetism is evolving into a thriving subfield of nanomagnetic research with a number of exciting theoretical and experimental results [5,6,7,8].

**Figure 1:**
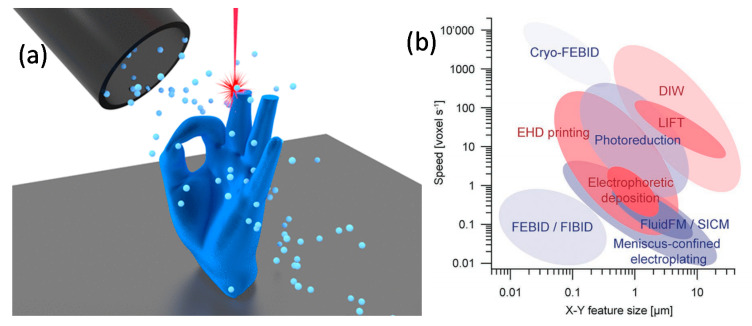
(**a**) Schematics of focused electron beam induced deposition (FEBID) for 3D nano-patterning, where gas injected by a nozzle is adsorbed on a substrate, with a fraction getting decomposed by a focused beam of electrons. By controlling the time the beam dwells on each point, 3D nano-printing of magnetic materials becomes possible. Reproduced with permission from [16]. Copyright 2020 American Chemical Society. (**b**) Comparison of speed and resolution of FEBID and its sister technique focused ion beam induced deposition (FIBID), with other emerging additive manufacturing methods for the direct writing of metallic micro- and nano- structures. FEBID at cryogenic temperatures (cryo-FEBID) enables direct-writing at very high growth rates, with slightly worse resolution [17,18]; a similar resolution as cryo-FEBID, with an approximately 100 increase in growth rate has been recently demonstrated using cryo-FIBID [19,20] (not shown here). Adapted with permission from [21].

Specifically, one of the key driving forces behind advances in the field is the development of 3D fabrication methods that push current resolution limits and control material properties, with, e.g., two-photon lithography [9,10,11] and electrodeposition [12,13,14] as two clear exponents of such methods. In this review, we focus on focused electron beam induced deposition (FEBID), an additive manufacturing technique that is quickly developing into a versatile tool to grow 3D nanomagnets and control their geometry with resolutions down to a few tens of nanometres [15], see Figure 1. 

Here, we describe some of the most important recent advances in the growth, characterization and application of magnetic nanostructures fabricated by FEBID for 3D nanomagnetism. The article is structured as follows: Section 2 discusses recent advances on the direct writing of complex 3D geometries via new computational frameworks and other approaches. Section 3 is devoted to the material properties of 3D FEBID ferromagnetic nanostructures, including ways to control the magnetic and crystallographic properties of single-element materials, as well as alloys. Section 4 includes a selection of recent works where 3D FEBID nanostructures are being used for different applications: from the well-consolidated area of scanning probe microscopy, to other emerging research areas such as magnetic frustration, chiral magnetism, magnonics and superconducting spintronics. Section 5 outlines conclusions and perspectives of FEBID for applications in 3D nanomagnetism.

## 2. Writing Nanomagnets Using FEBID

The fabrication of complex-shaped 3D nanomagnets by FEBID breeds from pioneering works where computational tools devoted to 3D nanofabrication using FIBID and FEBID were developed [22,23]. In the last few years, together with generic software exploiting in-built capabilities of standard 3D printing adapted to focused electron/ion tools [24], specialized FEBID patterning software has been developed for 3D printing at the nanoscale [16,25,26]. This has enabled the realization of a variety of 3D nanostructures, from artificial nanowire lattices [25,26] to shapes with curved surfaces and complex topologies [16], including in both approaches the successful usage of magnetic precursors [16,27]. We discuss here the main features of the FEBID process, highlighting key differences with respect to other 3D printing methods. We further describe the recent advances that are simplifying structure design and generation of beam scanning patterns, including the new efforts to calculate optimal patterns for general 3D structures directly from stereolithography (STL) files.

### 2.1. 3D Printing via FEBID

**Figure 2:**
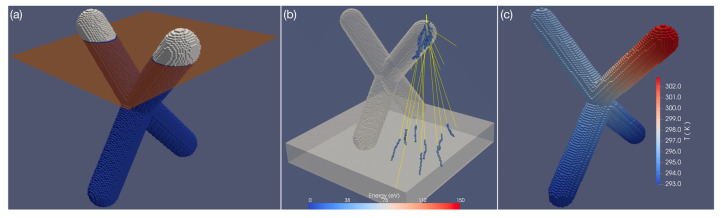
(**a**) Exemplary 3D computer-aided design (CAD) model of a ferromagnetic tetrapod structure with slicer plane for 2D pattern generation, for illustration. (**b**) Result of Monte Carlo simulation of several primary electron trajectories at 20 keV energy for tetrapod structure illustrating elastic scattering (yellow trajectories) and deposited energy (colour bar), due to inelastic scattering. (**c**) Temperature distribution in tetrapod structure under electron beam exposure at 20 keV and 44 pA beam current, when the electron beam hits at the topmost upper front arm (stationary state finite difference solution of heat conduction equation). The temperature increase is from 293 K at the base of the structure to about 303 K at the beam impact position; material parameters of Pt_20_C_80_.

Regarding the 3D FEBID process itself, several points must be considered. Starting from a 3D computer-aided design (CAD) model, a procedure must be developed that maps the 3D coordinates onto a sequence of 2D writing patterns, needed for a bottom-to-top fabrication of the target structure. Figure 2a schematically indicates a 2D pattern slice of a 3D CAD structure. However, the dynamics of the adsorbed precursor, as well as the trajectories of the scattered electrons from the primary beam, must be taken into account. In this regard, slicing a 3D CAD model for 3D FEBID is significantly harder than the analogue task known from the 3D printing of polymers. On the simplest level, the precursor dynamics and local growth rates during the writing process can be simulated by numerical solution of the diffusion-reaction equation on the evolving 3D surface [28]. This has to be coupled with considerations of the transferred beam energy in two accounts. First, scattering of primary, forward and backscattered electrons leads to dose deliveries which are not local to the primary beam impact position (see Figure 2b). At any point of the surface where energy is provided due to inelastic collisions, secondary electrons may leave the surface and trigger a precursor dissociation event causing growth. Second, the volumetrically deposited energy can lead to the heating of the growing 3D structure, which can become particularly severe for low-metal content deposits with associated low heat conduction. For deposits, e.g., from the well-known precursor Me_3_CpMePt(IV), this has been shown to be a major problem [29]. Figure 2c shows an example for local heating effects in the last growth stages of a tetrahedral target structure at typical beam energies for high-resolution growth. Here, the temperature increase can easily exceed 10 K during growth, which leads to an appreciable increase of thermal desorption of the precursor and thus to a reduced local growth rate [16]. In contrast to highly-carbon-based materials grown using non-magnetic FEBID precursors, magnetic 3D nanostructures are typically much higher in metallic content, normally reaching contents greater than 80 at.% (see Section 3). This helps to mitigate this heating problem and it may be neglected in many cases.

In the development of efficient pattern generation software, the issues of (i) height-dependent growth rate variation due to diffusion limits, (ii) precursor consumption due to proximity effects, (iii) height- and material-dependent heating effects and (iv) non-local deposition due to scattering, have to be accounted for. Presently, available patterning programs [16,25,26] take (i) and (ii) into account, and the one most recently developed [16] tackles partially (iii) by compensating for heating effects assuming the growth of a single-material with effective thermal conductivity. Non-local deposition (iv), however, remains an issue for future research, as a full simulation of 3D writing process [22] is likely to be necessary for a complete compensation of non-local deposition artifacts by suitable adaption of the 2D writing patterns.

### 2.2. 3D Printing of Arbitrary-Shaped Nanomagnets

Until quite recently, the main emphasis regarding 3D nanofabrication by FEBID has focused on high-purity nanowires and nanowire networks, due to their wide scientific and technological applicability [30]. However, FEBID can fabricate a much wider range of structures, having all the capabilities of a traditional extruder-based 3D printer, but with a several orders of magnitude higher resolution [31].

The final goal of FEBID 3D printing consists of the faithful reproduction of any 3D CAD model, while independently controlling the material properties, which is far from trivial when working at the nanoscale and using focused electron beams and gases. Complex electron interactions with the deposit and the substrate, together with a number of competing effects, such as gas flux, temperature and diffusion, are dynamically changing the local deposition rates during the fabrication [18,32]. Each of these effects is further dependent on a number of parameters, many of which are difficult to measure with the required level of certainty [15,29,32]. Extensive research is under way combining hybrid Monte Carlo-continuum simulations with experimental feedback to pinpoint the key parameters and to examine the deposition process in detail [22,25,29].

**Figure 3:**
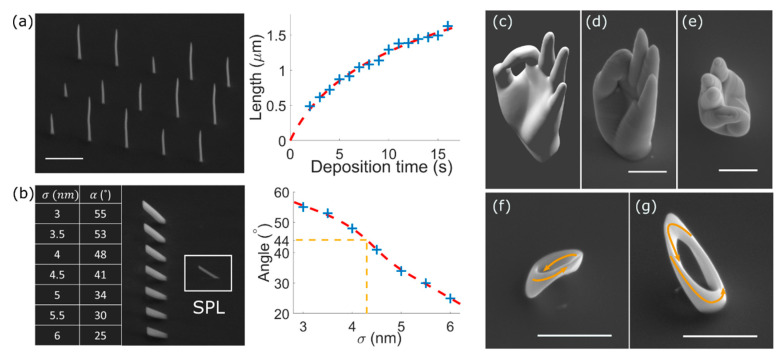
(**a**) An array of vertical pillars is built with varying deposition times. The height of the resulting structures is used to determine the base growth rate, and temperature scaling factor, reducing the growth rate as pillars get longer. (**b**) The effective standard deviation σ of the deposit is determined by comparing wide nanowires to a single pixel line (SPL), allowing correction for proximity effects. (**c**) Stereolithography (STL) model of a human hand. (**d**,**e**) Side and top view SEM images of the model fabricated with MeCpPt(Me)_3_. (**f**,**g**) Ferromagnetic Möbius strip made using Co_2_(CO)_8_, where arrows are included to help visualize the geometry. Scale bars are 1 µm. Adapted with permission from [16]. Copyright 2020 American Chemical Society.

However, despite this complexity, recent works show how, by carefully choosing the regime of growth, the large number of fundamental parameters can be reduced to a few effective ones that can be directly measured and are capable of modelling the deposition (see Figure 3a,b) [16,18]. This simplified model is computationally tractable, directly creating beam scanning patterns from STL files used in traditional 3D printers.

A range of complex geometries have been nano-printed using this approach (see Figure 3c–g), with both standard MeCpPt(Me)_3_ (that can be e.g., used as a scaffold for subsequent deposition of magnetic thin films, Section 3.3), and the magnetic precursor Co_2_(CO)_8_, demonstrating a promising route to fabricate general 3D magnetic nanostructures with FEBID. Further work, however, is necessary to generalize the model to other precursors and more complex regimes of growth where higher-purity materials can be achieved (Section 3).

## 3. Ferromagnetic 3D Nanostructured FEBID Materials

The happy marriage between FEBID and nanomagnetism is primarily due to the fact that room-temperature ferromagnetic materials can be directly grown with this technique under particular growth conditions [33,34,35]. Co_2_(CO)_8_, Fe(CO)_5_ and Fe_2_(CO)_9_ [36,37,38] are the most prevalent precursors used for this purpose, giving rise to either Co or Fe materials with metallic purities typically above 75 at.%. This contrasts with most other FEBID precursors, mostly designed for chemical vapor deposition (CVD) applications, where materials typically formed by ~70–80 at.% carbon are grown, primarily due to the contamination by organic groups present in the precursor [33] as well as contaminants originating from the dissociation of residual gases present in the vacuum chamber [39]. Investigations devoted to the development of new precursors is key for further progress in this area. However, stringent requirements for precursor properties make the discoveries of practically usable precursors difficult [40]. We discuss here recent advances regarding obtaining highly-pure Co and Fe 3D deposits, as well as new routes to integrate ferromagnetic alloys onto 3D nanostructures.

### 3.1. Tuning of Cobalt and Iron FEBID Materials by Post-Growth Annealing

As described above, a generally common issue regarding FEBID materials is the existence of chemical impurities incorporated into the main deposit, which may become limiting factors for some applications. To solve this fundamental limitation, diverse approaches such as ex situ [41] and in situ [42] thermal annealing and electron beam irradiation at high vacuum and under controlled reactive gas atmospheres [43,44], use of substrates at high temperatures [45,46] or post-growth Joule heating and electromigration upon injection of high electric currents [47] have been conducted in the past. Such strategies have been predominantly employed in non-magnetic materials, where the purity of FEBID materials is generally very low [33].

For ferromagnetic materials, post-growth purification methods [42] seem particularly appealing when fabricating 3D nanostructures, due to several reasons. For instance, there exists evidence of a drastic reduction of the metallic content in high aspect ratio FEBID nanowires with small diameters, due to complex temperature-dependent effects taking place during 3D FEBID [48]. Furthermore, a high surface-to-volume ratio may also result in the oxidation of a substantial fraction of this type of structures [49], having led, e.g., to strategies involving non-magnetic gases to protect them [50,51].

**Figure 4:**
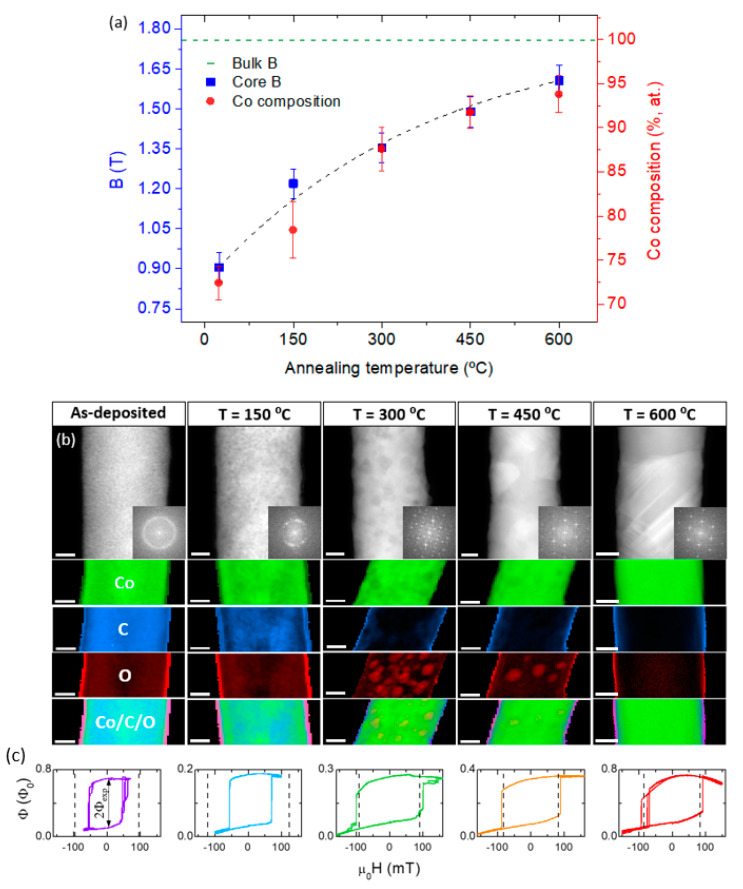
Scanning transmission electron microscopy characterization of Co nanowires under an annealing post-growth purification process. (**a**) Average B-field and Co composition as a function of annealing temperature measured by off-axis electron holography. (**b**) High-angle annular dark-field and corresponding fast-Fourier-transform (first row), and chemical maps by electron energy loss spectroscopy (following rows). Scale bars are 20 nm. (**c**) Hysteresis loops obtained by nano-SQUID (super quantum interference device) magnetometry at 15 K for each of the wires. (**a**,**b**) Reproduced with permission from [52]. (**c**) Reproduced with permission from [55]. Copyright 2018 American Chemical Society.

Recent works have employed post-growth thermal annealing under high vacuum conditions to tune the purity, crystallinity, magnetic induction in cobalt and iron FEBID free-standing cylindrical nanowires [52,53], revealing significant differences between both materials. In the case of Co (Figure 4), the study focused on 3D nanowires of ≈90 nm in diameter and an initial ≈75 at.% metallic content, which were subject to an in vacuum, ex situ post-growth annealing up to 600 °C. This thermal treatment increased the metallic content up to 95 at.% (Figure 4a), and at the same time induced the recrystallization of the pseudo-amorphous as-deposited structure into hcp and fcc crystals, with lateral crystal sizes comparable to the nanowire diameter (Figure 4b). This contrasts with the study performed on iron nanowires of ≈50 nm in diameter and an initial metallic content ≈40 at.% (not shown here), where similar annealing conditions revealed how the as-deposited homogeneous nanocrystalline Fe structure evolved during annealing into a material presenting a strong phase segregation, formed by a combination of (highly-pure Fe) metallic and (richer in C and O) amorphous regions. In this second case, the general morphology of the nanostructure was preserved.

Importantly, in the case of Co, the annealing process led to a net magnetization increase of 80% with respect to as-grown values (Figure 4a), up to 1.6 T, approaching the value of bulk Co. This procedure also showed a minor volume shrinkage effect, unlike in other FEBID materials [54], which led to good mechanical stability conditions during annealing and consequently to the original shape of the 3D nanowire being mostly maintained. The magnetic reversal of these nanowires was subsequently measured by nano-SQUID (superconducting quantum interference device) magnetometry [55], revealing that the enhancement of Co content and crystallinity under annealing results in larger magnetic switching fields and better-defined magnetic switching field values (Figure 4c).

### 3.2. Ferromagnetic Alloys

The integration of ferromagnetic alloys onto 3D nanostructures offers various advantages with respect to single-element materials regarding the fine-tuning of desired magnetic properties, such as magnetic hardness, saturation magnetization or Curie temperature [56]. It therefore comes as no surprise that several attempts have been made to fabricate alloy nanostructures by FEBID (see [57] for a more complete account). So far, three different approaches have been taken, namely (i) using two or three precursors in parallel [57,58,59], (ii) electron-beam or thermally induced intermixing of FEBID multilayer structures [60,61] and (iii) employing heteronuclear precursors [62,63].

Among these variants, the recent route of using a heteronuclear precursor has so far proved to be most successful, specifically with the carbonyl HCo_3_Fe(CO)_12_, which provides ≈80 at.% metallic Co_3_Fe deposits, it is easy to handle and is suitable for high resolution work at both, low and high beam energy [62]. It should be stressed, however, that this is not guaranteed if heteronuclear precursors are used. The chemically and structurally similar precursor H_2_Ru_3_Fe(CO)_13_ resulted instead in deposits with a maximum metal content below 30 at.% and was found to exhibit a distinctively different dissociation behaviour under low-energy electron impact [64]. At present, like in single-element (Co and Fe) FEBID materials, the most promising pathway to tap into the large reservoir of potential alloy precursors is the carbonyls. The presence of unoccupied orbitals with a dense band of ligand π* orbitals, mixed with metal centred orbitals, in conjunction with a dense constellation of occupied metal d-orbitals and associated band-like structure at the HOMO-LUMO gap of the bonding orbitals, may be the most important ingredient to obtain an effective FEBID process [64].

### 3.3. 3D FEBID Scaffolds and Magnetic Thin Films

**Figure 5:**
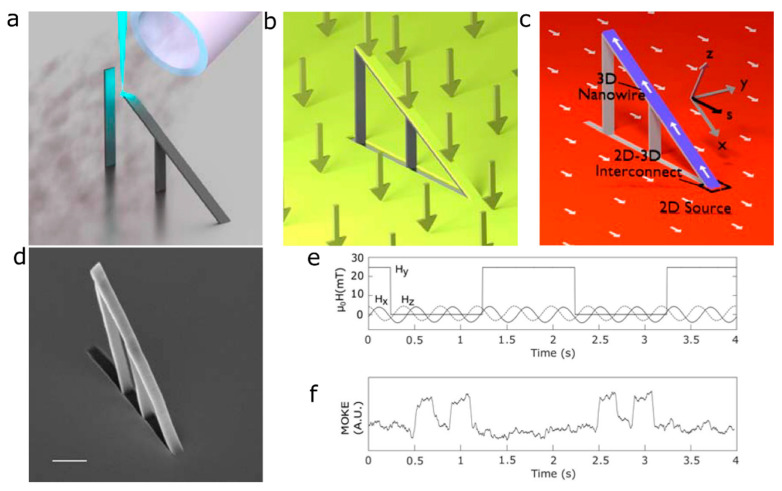
Fabrication and characterization of 3D FEBID-PVD (physical vapor deposition) hybrid nanostructures. (**a**) Non-magnetic FEBID scaffold fabricated with the MeCpPt(Me)_3_ precursor. (**b**) Permalloy evaporation onto the structure. (**c**) Schematic of the resulting nanomagnetic system. The 2D film acts as the source of domain walls which can be injected into the nanowire via the 2D-3D interconnect. (**d**) SEM image of the fabricated nanowire. Scale bar is 1 µm. (**e**) External magnetic fields applied as a function of time. The coordinate system is defined with x being along the length of the nanowire and y being parallel to the film (see **c**). A transverse field (H_y_) (is employed as a magnetic gate to control the injection of domain from the film transmitted by the rotating H_x_ and H_z_ fields. (**f**) Magnetic switching of the nanowire via domain wall motion, probed by dark-field magneto-optical Kerr effect. Adapted with permission from [65]. Copyright 2017 American Chemical Society.

Together with the direct writing of ferromagnetic materials, a hybrid approach combining 3D nano-printing by FEBID of non-magnetic scaffolds and a subsequent physical vapor deposition (PVD) method of magnetic thin films has been recently developed [65,66], see Figure 5a–d. This approach offers the key advantage of integrating into a 3D geometry highly-pure thin film spintronic materials, eliminating possible issues due to the lack of purity and limited number of ferromagnetic materials currently available by FEBID. It is, however, subject to issues such as non-conformality deposition of PVD processes, shadowing effects, and the deposition of the magnetic material on the whole wafer, around the 3D nanostructure.

This approach has been successfully followed by using the standard MeCpPt(Me)_3_ precursor to fabricate 3D nanowire scaffolds, followed by the PVD of a thin layer of magnetically-soft permalloy film, with the nanowire smoothly connected to the planar substrate [65]. The domain wall motion in these devices was measured by optical means, using a new dark-field magneto-optical method that is capable of probing the magnetic properties of a 3D nanostructure surrounded by a magnetic substrate. Due to the nanowire and substrate being at different planes, these structures can exhibit a significantly more complex response to vector external magnetic fields than their 2D counterparts. Specifically, domain walls can be controllably generated in the substrate plane, pinned at the 2D-3D interconnect region, and injected into the nanowire. The injection can further be gated by applying a field transverse to the nanowire (Figure 5e,f).

## 4. FEBID Nanostructures for 3D Nanomagnetism

Given the unique capabilities of FEBID for the additive manufacturing of metals at resolutions comparable with the magnetic length scales (Section 1) and the recent advances in 3D nano-printing (Section 2) and integration of ferromagnetic materials (Section 3), there are multiple areas of nanomagnetism where FEBID nanostructures may play a crucial role to explore new physical effects in the coming years [5,8]. Here, we discuss recent works in some areas identified as particularly promising.

### 4.1. Scanning Probe Microscopy Magnetic Sensing

Scanning probe microscopy (SPM) is one of the key applications where FEBID has excelled since its invention, thanks to its ability for 3D nanofabrication at almost any location, which offers unique opportunities for applications where specialized shapes are needed [33,67].

**Figure 6:**
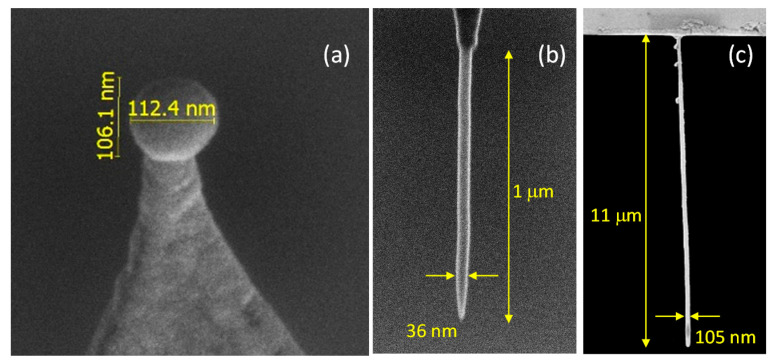
SEM micrographs of the three types of magnetic sensors grown by FEBID on cantilevers for scanning probe microscopy. (**a**) Co nanosphere on a cantilever for ferromagnetic resonance force microscopy. (**b**) Fe tip for magnetic force microscopy. (**c**) Long Co nanowire for scanning magnetic force sensing. (**a**) Reproduced with permission from [71]. (**c**) Reprinted with permission from [73]. Copyright 2020 by the American Physical Society.

In the realm of magnetic SPM, three different types of FEBID sensors have been reported so far. The first type consists of small magnetic tips grown on cantilevers for ferromagnetic resonance force microscopy (FMRFM), a technique dedicated to image the spin dynamic properties of a magnetic sample. In ferromagnetic resonance (FMR), the application of an a.c. magnetic field perpendicular to the sample magnetization direction can excite its ferromagnetic resonance, an effect that gets modified due to the force between the magnetic tip and sample in FMRFM. This technique is useful to investigate the spatial dependence of the ferromagnetic resonance modes in a magnetic nanostructure, as well as the magnetic coupling in between nearby magnetic nanostructures [68]. The lateral resolution of the technique depends on the size of the magnetic tip, which has led to the development of magnetic tips based on sub-micron probes. For instance, the ability of FEBID to grow small Co magnetic tips with diameters down to 10 nm has been exploited to carry out high-resolution FMRFM measurements [69,70]. Moreover, FEBID has also been used to grow Co nanospheres as small as 100 nm in diameter [71], as shown in Figure 6a. The spherical shape is preferred for this type of application, since it minimizes possible magnetic hysteresis effects, making the quantitative analysis of the measurements simpler [72].

The second type of SPM sensor by FEBID concerns conventional magnetic force microscopy (MFM). In MFM, a magnetic tip at the edge of a cantilever is used to study the magnetic state of a sample through the modification of the cantilever resonance frequency, an effect that occurs due to the change of the tip-sample magnetic force during a scan. In general, magnetic tips are created by covering one side of standard atomic force microscopy (AFM) tips with a few-nm-thick magnetic film, typically by sputtering, an approach that provides magnetic contrast but which limits the spatial resolution of the method, due to the pyramidal shape of the tip. Instead, the use of FEBID makes the growth of high aspect-ratio magnetic tips possible (Figure 6b), providing significantly better magnetic resolution [37,74,75]. FEBID magnetic tips are of particular interest in the case of cantilevers based on either piezoelectric or resistive detection, as is sometimes the case for stringent conditions such as low-temperatures or high vacuum [76]. Moreover, recent results report how FEBID magnetic tips shaped as small-diameter nanorods are beneficial to produce confined magnetic stray fields, enhancing magnetic sensitivity and facilitating quantitative data analysis [77]. This type of FEBID nanorod magnetic tips present higher coercive fields than standard ones, being less affected by magnetic stray fields emanating from the sample, as well as external magnetic fields. Furthermore, they can be customized to produce low magnetic stray fields, which is necessary for MFM studies of spin textures in magnetically-soft samples [78]. Recent results also indicate that FEBID MFM tips present a great performance under liquid environment, opening new routes in bio-magnetics [77,79].

The third type of application of magnetic FEBID nanostructures for SPM concerns its use in scanning magnetic force sensing (SMFS), as demonstrated in [73]. Very high aspect-ratio Co nanowires with lengths ≈11 µm and ≈100 nm in diameter were found in this publication to be excellent probes for this technique. In SMFS, changes on resonance frequency associated to nanowire’s flexural vibration modes, due to forces between the sample and the nanowire sensor, are exploited to image a magnetic sample. The Co FEBID nanowires used for this purpose were found to be well-behaved mechanically, with high quality factors of up to 2000. In this dynamic cantilever magnetometry technique, the superb sensitivity obtained, equal to 3 nT/Hz^0.5^, is comparable to some of the most sensitive scanning probes available [80], including scanning nitrogen-vacancy magnetometers and scanning SQUIDs. In contrast to MFM, here the sample’s stray field interacts only with the magnetic charge distribution at the very end of the nanowire close to a sample. This leads to a superior sensitivity and enables to work in a regime of low invasiveness. The technique is also promising to develop SPM methods with 3D vector magnetic force sensing capabilities.

### 4.2. Magnetic Nanowires and Nanowire Networks

Nanowire-based structures have been the dominating geometry targeted by FEBID, see, e.g., [36,37,81,82], even before the advent of systematic and simulation-assisted 3D FEBID. In magnetism, 3D nanowire structures, as well as 3D networks formed by repeating nanowire-like unit cells, are very attractive. 3D FEBID single nanowire structures can be used for a range of applications, from sensors in scanning probe microscopy (Section 4.1) and domain wall conduits (Section 3.3), to field-driven nano-actuators [83], to cite a few. Furthermore, 3D geometries open up exciting perspectives to explore new theoretically-predicted effects exploiting the interplay of nanoscale geometry and curvature with the magnetization (Section 4.2).

**Figure 7:**
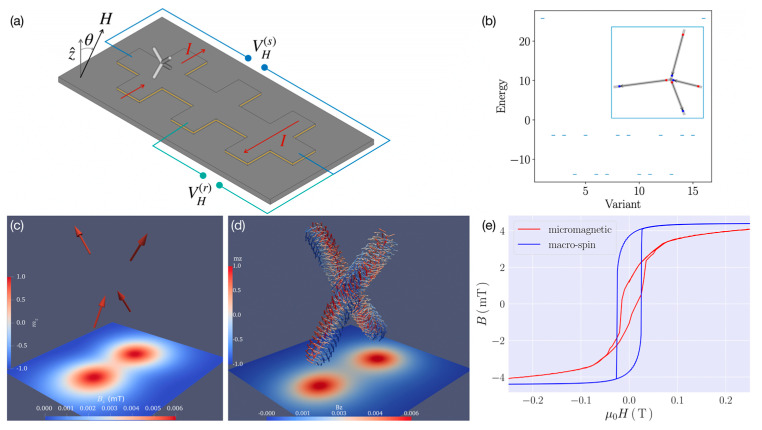
(**a**) Schematic of typical gradiometry setup for micro-Hall magnetometry with a tetrapod 3D ferromagnetic structure fabricated by FEBID; see [85] for details. From the measured Hall voltages V_H_^(s)^ and V_H_^(r)^, the magnetic stray field generated by the tetrapod can be deduced. (**b**) Energy diagram of a tetrapod structure assuming uniformly magnetized arms within the dumbbell approximation. The sixfold degenerate ground state refers to the “two in – two out” ice rule for tetrahedral spin ice [86]. Configuration #4 shown in the inset depicts one of the possible ground states. (**c**) Macrospin model for the tetrapod with uniaxial anisotropy along the arm directions in zero field, where a “two in–two out” state is realized. (**d**) Result of zero-field micromagnetic simulation of tetrapod structure using mumax^3^ [85] with material parameters of Co_3_Fe; see [85] for details. The colour code on the plane of the 2DEG represents the z-component of the magnetic stray field generated by the tetrapod. (**e**) Comparison of results of stray field calculations for Co_3_Fe tetrapod with field perpendicular to the 2DEG. Micromagnetic (red) and macro-spin (blue) simulation show roughly corresponding coercive fields but clear differences in details of the stray field hysteresis. Suitable parameter selection for the macro-spin simulation can reduce these differences to some degree; see [28] for details.

Furthermore, 3D nanowire networks are highly significant with regard to realizing extended magnetically frustrated systems on the meso-scale [84], as recently investigated via 3D FEBID Co_3_Fe tetrahedra by means of micro-Hall magnetometry. In these experiments, the magnetic stray field generated from a tetrahedra grown within the sensor area of a 2D electron gas (2DEG) semiconductor heterostructure was measured by means of the Hall voltage generated within the 2DEG (Figure 7a). Conceptually, such a tetrahedral building block consisting of uniformly magnetized cylindrical arms exhibits a sixfold degenerate magnetic ground state (Figure 7b). If extended into a 3D diamond-like lattice, this represents an artificial spin ice equipped with several tuning options, such as saturation magnetization and strength of dipolar coupling, something achievable in FEBID by diluting the magnetic component via the mixture with other precursor, as well as by scaling the lattice constant. In this case, however, 3D cylindrical structures of 60–70 nm in diameter made vortex-like local magnetization distributions energetically favored [85]. Therefore, whereas a simple macro-spin model could reproduce concise features in some of the measured magnetic hysteresis curves, these were reproduced more quantitatively via full micromagnetic simulations [27] (see Figure 7c–e). The magnetic frustration effects associated to these curvature-induced magnetization structures is a work in progress.

### 4.3. Curvilinear Nanomagnetism

Magnetism in curvilinear geometries has emerged as a rapidly developing domain of modern magnetism with many exciting theoretical predictions and strong application potential [6,87,88]. By engineering the 3D shape and local curvatures, the intrinsic magnetic couplings can be modified, which has allowed for the predictions of magneto-chiral effects [89], topologically induced magnetization patterning [90], absence of the breakdown velocity for domain walls [91], chirality symmetry breaking [87,92] and Cherenkov-like [93] magnonic effects. Among other geometries, magnetic nanotubes and nanowires are those best explored so far, due to the range of fabrication techniques able to create these simpler geometries [94]. However, the lack of suitable 3D nanofabrication techniques has held back experimental studies in more complex geometries such as tori, Möbius strips, and spherical shells. We anticipate that CAD-assisted direct-write of 3D FEBID nano-architectures (Section 1) should bridge this gap between theory and experiments in the future, opening new horizons for curvilinear magnetism.

**Figure 8:**
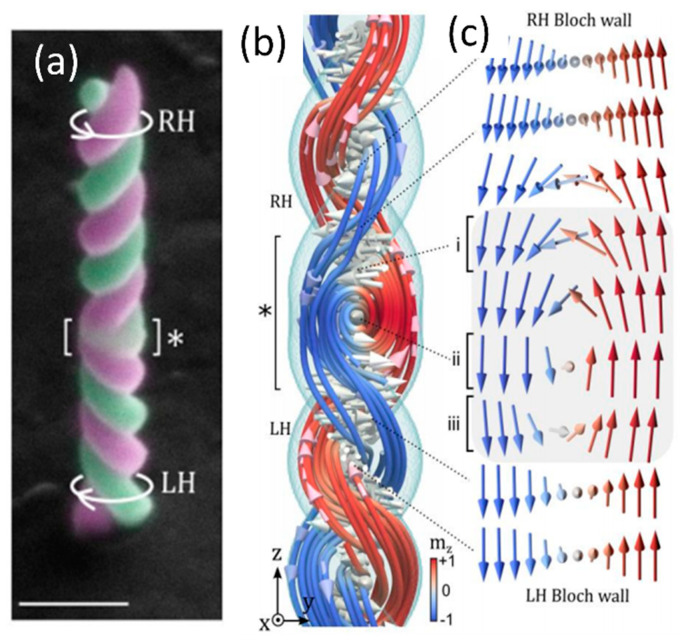
(**a**) Double-helix with opposite chirality (bottom LH: left-handed; top RH: right-handed) interfaced at the region marked by an asterisk. Scale bar is 1 µm. (**b**,**c**) Micromagnetic simulations of the double-helix system in an antiparallel magnetic state; a Bloch wall with a well-defined chirality is formed between the strands, with the chirality defined by the chirality of the corresponding helix. A 3D vortex with a Néel defect is formed at the region (*) connecting both chiralities. Reproduced with permission from [98]. Copyright 2020 American Chemical Society.

A complex chiral geometry that is particularly appealing in this realm is the helix, where control over diameter, curvature and torsion at the nanoscale can lead, via an effective Dzyaloshinskii-Moriya interaction, to exotic magnetization distributions and a very rich phenomenology [95]. This type of geometry has been successfully obtained by FEBID in the past for both non-magnetic [96] and magnetic [36] precursors, with recent results combining electron off-axis holography and tomography to characterize Co helices spanning a range of curvature and torsion values [97]. In addition, the 3D writing ability of FEBID has been just exploited to a greater extent, by interfacing two double helices with strongly-overlapped strands of opposite chiralities [98], see Figure 8a–c. This approach, as demonstrated by transmission X-ray magnetic microscopy and micromagnetic simulations, can be exploited to imprint chiral spin states via geometrical chirality only; it also enables the formation of localized complex 3D spin textures and topological defects at regions mediating the transition between geometrical chiralities, something arguably not possible in standard approaches using bulk and thin film magnetic systems.

### 4.4. Superconducting Spintronics and Fluxonics

Hybrid systems composed of superconductors (S) and ferromagnets (F) harbor numerous physical phenomena emerging due to the antagonistic spin ordering [99] that affects spin transport [100] and dynamics of magnetic moment excitations [101]. Examples are odd-frequency spin-triplet superconductivity [102] favoring non-collinear magnetization environments [103] and leading to long-range proximity [104,105], giant thermoelectric [106] and thermo-spin [107] effects. In addition, S/F heterostructures find applications in magnetic recording [108], information storage devices [109], and magnetic cloaking metamaterials [110]. Inhomogeneous magnetization configurations induced by the geometry or topology of 3D FEBID structures should thus be pivotal for engineering new states of matter in which the low-dissipative response of S is combined with the magnetic order of F, opening new horizons for quantum computing.

**Figure 9:**
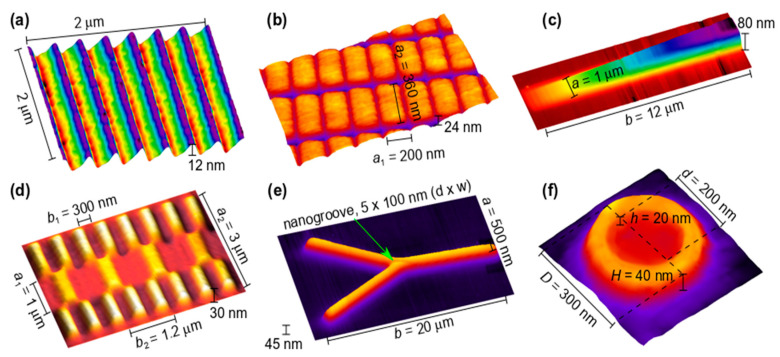
Atomic force microscopy images of exemplary FEBID structures for fluxonics and magnonics. (**a**) Nb film decorated with an array of asymmetrically shaped Co nanostripes inducing a ratchet pinning potential landscape of the washboard type for Abrikosov vortices. (**b**) Bi-periodic magnonic crystal on the surface of a Py film that allows for reprogramming the band structure in the magnon frequency spectrum. (**c**) Magnonic waveguide with a gradually decreasing thickness that induces a graded refractive index for spin waves via the magnetization gradient. (**d**) 3D magnonic crystal in which the thickness modulation period is a factor of two larger than the width modulation period. (**e**) Y-shaped magnonic waveguide with a nanogroove milled by focused ion beam (FIB) at the junction for frequency-selective steering of spin waves via the refraction and reflection effects. (**f**) “Nano-volcano” for ferromagnetic resonance studies. Structures (**b**–**f**) are fabricated from Co_3_Fe employing the precursor HFeCo_3_(CO)_12_.

Ferromagnetic “decoration” of superconducting films provides a traditional means to induce tailored pinning potentials which influence the dynamics of Abrikosov vortices [110,111,112,113,114], see Figure 9a,b. Recently, Co-FEBID nanostrips extended into the third dimension by shaping their cross-section in an asymmetric fashion (Figure 9a) has allowed for breaking the symmetry of the vortex motion under current polarity reversal and studying vortex ratchet (rectification) effects [115]. By guiding magnetic flux quanta at a small tilt angle with respect to a Co nanostrip array, about 5 km/s vortex velocities have been achieved [116] providing access to studying Cherenkov-like generation of acoustic [117] and spin [118] waves. We anticipate that 3D FEBID structures will also find applications in the rapidly developing field of magnon fluxonics [119,120,121,122], addressing the interplay of superconductivity and spin-wave physics. In addition, the suitability of FEBID for the fabrication of 3D leads should enable magneto-resistance measurements in both in-plane and out-of-plane current geometries [123,124,125].

A significant improvement of the microwave radiation detection has been demonstrated through the use of superconducting bolometers made from rolled-up planar microstructures into 3D helices [126]. At the same time, superconducting FEBID structures remain so far limited to the systems Mo-C-O (precursor Mo(CO)_6_, *T*_c_ ≈ 10 K) [127], W-C-O (precursor W(CO)_6_, *T*_c_ ≈ 2 K) [128], and Pb-C-O (precursor Pb(CH_3_CH_2_)_4_, *T*_c_ ≈ 7.3 K) [129]. While current work is directed at the synthesis of novel precursors for superconducting FEBID materials, 3D writing strategies are being extensively tested for the complementary technique of FIBID. Employing a He^+^ ion beam microscope, Córdoba et al. fabricated free-standing hollow superconducting tungsten carbide nanowires with diameters smaller than 32 nm [130] and 3D nano-helices with diameters of 100 nm [131]. In contrast to planar superconductor structures, the complex 3D geometry of nano-helices leads to topologically non-trivial screening currents and confinement potentials that depend on the curvature and torsion of the helices and stipulate the occurrence of different patterns of topological defects whose dynamics affects the resistive response [126,131]. In this way, once available, superconducting 3D FEBID structures will offer much potential for studying novel physics of geometry- and topology-induced effects [132,133], as well as for remote sensing and electronic applications.

Recently, a comparative study of structural and transport properties was reported for free-standing 3D nanowires fabricated by Ga^+^ FIBID and FEBID employing the precursor Nb(NMe_2_)_3_(N-t-Bu) [134]. Electrical transport measurements showed that FEBID nanowires are highly resistive, whereas FIBID planar nanowires become superconducting at *T*_c_ ≈ 5 K. Interestingly, the critical temperature of free-standing 3D nanowires is as high as *T*_c_ ≈ 11 K, which is close to the value of bulk NbC. Remarkably, Nb-C-FIBID exhibits a rare combination of properties: weak volume pinning, close-to-depairing critical current and fast heat removal from heated electrons. This provides access to investigations of vortex dynamics at >10 km/s vortex velocities [135] and renders Nb-C-FIBID as a candidate material for single-photon detectors, with properties comparable to NbN and MoSi thin films [136,137]. In addition, the direct-write capability of FIBID and FEBID should be fortunate for on-chip and on-fiber detector integration in circuits for quantum information processing.

### 4.5. Magnonics

Magnonics has emerged as one of the most rapidly growing research fields in magnetism [138]. It is concerned with the dynamics of spin waves, which are precessional excitations of ordered spins in magnetic materials. Covering a wide frequency range from sub-GHz to tens of THz and being free from the translational motion of electrons and the associated Joule heat, spin waves possess great potential for realizing novel, highly efficient wave-based computing concepts [139]. In this regard, FEBID, whose potential for magnonic applications has been demonstrated in a few proof-of-concept experiments [71,140,141], can offer unique features which go beyond the rich instrumentation of traditional fabrication techniques employed in magnonics.

Artificial magnetic media with properties periodically varied in space—magnonic crystals—are especially valuable for controlling and manipulating spin waves [142]. The spectra of spin waves in these materials exhibit forbidden-frequency regions (bandgaps), where spin waves are not allowed to propagate [139]. Of especial interest are bi-component, reprogrammable magnonic crystals whose magnetic configuration can be switched between different states [142]. Such crystals can readily be fabricated by using a combination of FEBID with focused ion beam (FIB) milling, with a unique possibility to gradually modify the spin-wave transmission characteristics by stopping the deposition/milling process at the desired stage, or continuing it after a magneto-dynamic measurement.

One important function of future spin wave-based computers is to control their propagation. The challenge of steering spin waves has primarily been addressed in curved waveguides, due to losses and scattering in their bends. An alternative solution is to steer spin waves via a graded refractive index, which smoothly alters the wave trajectory with minimal reflections [49]. To achieve a graded index for spin waves, one must gradually change a magnonic parameter (e.g., magnetization), which was demonstrated to steer spin waves around a 90° corner [143] and suggested for the development of graded-index magnonic fibers and lenses [144]. A variation of the magnetization in FEBID structures can be achieved by choosing specific writing strategies, beam parameters, and/or post-growth irradiation of structures with ions or electrons. An example of a graded-index magnonic conduit is presented in Figure 9c.

The capability to fabricate 3D nano-architectures appears to be the strongest advantage of FEBID. Previous experiments on nanodisks with nanoholes [145] revealed that their magneto-dynamic response differs essentially from that of flat ones. As conventional complementary metal–oxide-semiconductor (CMOS) technology becomes three-dimensional and magnonics is aimed at remaining at the same technological level, magnonic networks are also extended into the third dimension [146]. Of particular interest here are 3D nanoresonators [147], 3D magnonic crystals [147], as well as directional couplers [148] and frustrated magnetic systems [149,150,151,152] whose extension into the third dimension is expected to significantly enhance their functionality. Thus, while frustrated 3D magnetic nanowire lattices fabricated by two-photon lithography have recently been demonstrated [153], further optimization of 3D nano-cube and nano-tree FEBID lattices [27,85] towards higher metal contents and—ideally—a single-domain state of these building blocks should allow for building magnonic 3D nano-architectures with complex interconnectivity and for the development of novel types of human brain-inspired neuromorphic magnonic networks. An example of a 3D magnonic crystal prepared by FEBID is shown in Figure 9d, while examples of a magnonic waveguide joint and a 3D “nano-volcano” structures are presented in Figure 9e,f.

## 5. Conclusions and Perspectives

We have discussed key advances of 3D magnetic nanostructures grown by FEBID in the last few years. Fundamentally, the recent advances in understanding of competing fundamental effects during FEBID deposition lead to the development of new pattern generating software for 3D nano-printing, opening exciting opportunities in a range of nanotechnology areas. The growth of room-temperature ferromagnets with tunable purity and magnetic properties makes FEBID particularly suitable for nanomagnetism. The unique performance of the technique to direct writing of ferromagnets with complex 3D shapes, in combination with the maturity reached by the technique, as exemplified by the works described here, opens exciting opportunities in a variety of areas in the emerging field of 3D nanomagnetism.
